# BPSL1626: Reverse and Structural Vaccinology Reveal a Novel Candidate for Vaccine Design against *Burkholderia pseudomallei*

**DOI:** 10.3390/antib7030026

**Published:** 2018-07-19

**Authors:** Riccardo Capelli, Claudio Peri, Riccardo Villa, Arnone Nithichanon, Oscar Conchillo-Solé, Daniel Yero, Paola Gagni, Marcella Chiari, Ganjana Lertmemongkolchai, Marina Cretich, Xavier Daura, Martino Bolognesi, Giorgio Colombo, Louise J. Gourlay

**Affiliations:** 1 Istituto di Chimica del Riconoscimento Molecolare, Consiglio Nazionale delle Ricerche, Via Mario Bianco 9, 20131 Milano, Italy; riccardo.capelli@outlook.com (R.C.); claudio.btech@gmail.com (C.P.); paola.gagni@icrm.cnr.it (P.G.); marcella.chiari@icrm.cnr.it (M.C.); marina.cretich@icrm.cnr.it (M.C.); 2 Center for Complexity and Biosystems and Dipartimento di Fisica, Università degli Studi di Milano and INFN, 20133 Milano, Italy; 3 Computational Biomedicine Section, Institute of Advanced Simulation IAS-5 and Institute of Neuroscience and Medicine INM-9 Forschungszentrum Jülich, 52425 Jülich, Germany; 4 Department of Biosciences, Università degli Studi di Milano, Via Celoria 26, 20133 Milano, Italy; riccardo.villa@gmail.com (R.V.); martino.bolognesi@unimi.it (M.B.); 5 Center for Research and Development of Medical Diagnostic Laboratories (CMDL), Faculty of Associated Medical Sciences, Khon Kaen University, Khon Kaen 40002, Thailand; arnone.nithi@gmail.com (A.N.); ganja_le@kku.ac.th (G.L.); 6 Institute of Biotechnology and Biomedicine (IBB), Universitat Autònoma de Barcelona (UAB), 08193 Bellaterra, Spain; ocs@bioinf.uab.es (O.C.-S.); daniel.yero@gmail.com (D.Y.); xavier.daura@uab.cat (X.D.); 7 Catalan Institution for Research and Advanced Studies (ICREA), 08010 Barcelona, Spain; 8 Pediatric Clinical Research Center “Romeo ed Enrica Invernizzi”, Cryo Electron-Microscopy Laboratory, Università degli Studi di Milano, 20133 Milano, Italy; 9 Department of Chemistry, Università di Pavia, 27100 Pavia, Italy

**Keywords:** *Burkholderia*, BPSL1626 antigen, melioidosis, reverse vaccinology, crystal structure, in silico epitope predictions, type I fimbrial subunit

## Abstract

Due to significant advances in computational biology, protein prediction, together with antigen and epitope design, have rapidly moved from conventional methods, based on experimental approaches, to in silico-based bioinformatics methods. In this context, we report a reverse vaccinology study that identified a panel of 104 candidate antigens from the Gram-negative bacterial pathogen *Burkholderia pseudomallei*, which is responsible for the disease melioidosis. *B. pseudomallei* can cause fatal sepsis in endemic populations in the tropical regions of the world and treatment with antibiotics is mostly ineffective. With the aim of identifying potential vaccine candidates, we report the experimental validation of predicted antigen and type I fimbrial subunit, BPSL1626, which we show is able to recognize and bind human antibodies from the sera of *Burkholderia* infected patients and to stimulate T-lymphocytes in vitro. The prerequisite for a melioidosis vaccine, in fact, is that both antibody- and cell-mediated immune responses must be triggered. In order to reveal potential antigenic regions of the protein that may aid immunogen re-design, we also report the crystal structure of BPSL1626 at 1.9 Å resolution on which structure-based epitope predictions were based. Overall, our data suggest that BPSL1626 and three epitope regions here-identified can represent viable candidates as potential antigenic molecules.

## 1. Introduction

Melioidosis is an infectious disease caused by the Gram-negative, intracellular bacterium *Burkholderia pseudomallei* that resides in the soil of affected countries, predominantly in the subtropical and tropical regions of the world [1]. Melioidosis impacts heavily on affected populations, and mortality rates due to fatal septicemia have been reported to be as high as 50% and 19%, in North Thailand and North Australia, respectively [2]. Underreporting of the incidence of disease coupled to the inefficacy of antibiotic treatment has led recent research efforts in the direction of alternative therapies, namely a vaccine. In addition to vaccines that are based on live-attenuated or killed bacteria, capsule polysaccharides and several subunit components have been tested for their ability to induce immune protection in vivo [3]. Protein subunits that have been tested in vivo include flagellin subunits, outer membrane proteins, and a combination of chronic phase antigens [3,4,5,6,7].

For antigen discovery, reverse vaccinology involving whole genome screening of multiple pathogen strains or species for core-genome antigens is a rapid, safer, and cost-effective alternative to conventional antigen identification based on pathogen cultivation [8]. Antigen targets are selected, for example, based on the presence of signature sequences (signal peptides, transmembrane domains, etc.) that indicate their cell-surface location, the presence of MHC I/II binding sequences, or based on sequence homology with known antigens.

We demonstrate here the application of an in silico reverse vaccinology approach for the identification of a panel of 104 potential *B. pseudomallei* antigens. We show that BPSL1626, which is a predicted antigen and Type I fimbrial subunit, induces T-cell responses in vitro and that it is recognized by serum antibodies from human patients harboring diverse *Burkholderia* infections.

Furthermore, we report the crystal structure of recombinant BPSL1626 solved at 1.9 Å resolution, revealing a dimeric quaternary structure, comprising two monomers that possess the canonical, incomplete immunoglobulin-like fold of the Type-I fimbrial subunits. Structural and sequence information were next exploited to identify possible antibody interaction zones, employing different computational epitope predictors, based on independent physico-chemical principles to obtain a reliable consensus. The results that are presented here have the potential to define novel potential biomolecules for the development of multicomponent melioidosis vaccines targeting both arms of the immune system.

## 2. Materials and Methods

### 2.1. Reverse Vaccinology-Based B. pseudomallei Antigen Prediction

Detection of putative orthologs was carried out using a complete reciprocal best hits algorithm and UCSC blat as a comparison tool, accepting as ortholog groups, protein sets that have a complete graph associated to them, as previously reported [9,10]. The complete proteomes used in the initial comparative analysis of *B. pseudomallei* and *B. thailandensis* were downloaded from the last release of the integr8 database (ftp://ftp.ebi.ac.uk/pub/databases/integr8) [11]. For all other analyses, sequences were downloaded from the RefSeq ncbi genome database [12]. Cellular location was predicted using PSORTb [13]. HLA class I and class II binding predictions were performed with NetMHCpan and NetMHCIIpan, respectively [14,15], using alleles that are more frequently found in south Asian populations, according to dbMHC and AFND databases [16,17] (A*24:02, A*11:01, A*33:03, A*02:07:01, A*24:02:01, B*40:01, B*46:01:01, B*13:01:01, C*07:02, C*03:04:01:01, C*01:02, C*08:01, C*03:03 alleles were used for HLA class I and DRB1*14:01, DRB1*12:02:01, DRB1*08:03:02, DRB1*11:01:01, DQB1*03:01, DQB1*05:02, DQB1*05:01, DQB1*03:03:02:01, DQB1*02:01, DPB1*05:01:01, DPB1*04:01, DPB1*13:01, and DPB1*28:01 were used for HLA class II). Detection of remote homologs was performed with a hmm profile generated with jackhmmer using the BPSL1626 sequence to query a non-redundant database, created with CD-HIT, of UniProt bacteria, and using this hmm to search with the hmmsearch tool of the HMMER package in all previously downloaded proteomes [18,19,20]. The best hit for each genome, with similar score, *e*-value, and aligned positions as those that were obtained for BPSL1626 when searching blindly against *B. pseudomallei K96243*, was taken as a possible remote ortholog.

### 2.2. Cloning BPSL1626

The BPSL1626 gene (Uniprot code Q63UH6), coding for amino acid residues to 26–176 (minus the signal peptide) was amplified from *B. pseudomallei* strain K96423 genomic DNA (kindly provided by Prof. R. Titball, University of Exeter, UK) by PCR using the following primers: 1626-F:5′-CACCCAGACCGCGACGACCGGC–3′ and 1626-R:5′-CTACTTGTACGTCAGCGCGAATACCGC–3′ and Phusion DNA polymerase (Thermo Scientific), according to the manufacturer’s protocols. The gene was inserted into pET151/D-TOPO (Invitrogen), according to standard protocols. Successful cloning was confirmed by sequencing (Eurofins, Luxembourg).

### 2.3. BPSL1626 Expression and Production

BPSL1626 lacking the first 25 N-terminal residues encoding for the signal peptide was cloned in pET151/D-TOPO and overexpressed in *Escherichia coli* BL21(DE3)Star cells (Invitrogen, Carlsbad, CA, USA). For overexpression, cultures were grown in Luria Broth at 37 °C until an OD^600nm^ of 0.7–0.8 was reached. Upon cooling to 20 °C, induction was initiated by the addition of 0.1 mM IPTG. After overnight growth, cells from 0.5 L culture were harvested by centrifugation and resuspended in 50 mL Profinia IMAC wash buffer 1 (50 mM potassium dihydrogen phosphate pH 8.0, 0.3 M potassium chloride (KCl), containing 5 mM imidazole), containing lysozyme (0.20 mg/mL), DNases (20 μg/mL), and 10 mM MgCl_2_. Cells were mechanically lyzed at 25 mPa using a Constant Cell Disruption System (Constant Systems Ltd., Daventry, UK) and centrifuged at 16,000× *g* for 20 min and the soluble fraction was loaded onto a 5 mL Bio-Scale Mini Profinity IMAC cartridge (Biorad, Hercules, CA, USA), pre-equilibrated with IMAC wash buffer 1. The protein was washed with IMAC wash buffer 2, containing 10 mM imidazole and eluted with IMAC Elution Buffer, containing 250 mM imidazole. Fractions containing pure protein were pooled, concentrated, and exchanged into crystallization buffer (10 mM HEPES pH 7.0) using PD10 desalting columns. For antibody production, the His-tag was cleaved off by proteolysis with AcTEV protease (Thermo Fisher Scientific, Waltham, MA, USA), incubating overnight at 4 °C in the provided buffer. Cleaved protein was exchanged into 1X PBS.

### 2.4. Crystallization of BPSL1626

Crystallization screens of BPSL1626 (25 mg/mL) were set up in 96-well Greiner sitting drop plates, containing 100 μL of PACT-Premier crystallization screen (Molecular Dimensions) solutions in the reservoir and 400 nL drop volumes. Crystals grew after approx. one week at 20 °C in condition A9 (25% (*w*/*v*) PEG 6 K, 0.2 M lithium chloride, 0.1 M sodium acetate pH 5.0) and were cryoprotected prior to cryo-cooling in A9 supplemented with 30% (*v*/*v*) ethylene glycol.

### 2.5. Data Collection, Processing, Molecular Replacement, Model Building and Refinement

X-ray diffraction data at a resolution of 1.9 Å were collected on the BM-14 beamline (ESRF, Grenoble, France). Data were integrated with XDS [21,22] and assigned to the centered orthorhombic C222_1_ space group and scaled using POINTLESS and SCALA, respectively [23]. The structure was solved by molecular replacement using BALBES [24].

The crystal structure was refined to convergence (final R_cryst_ = 20.9% and R_free_ = 23.7%) while using Phenix.refine and structure geometry was validated using molprobity under the PHENIX platform (Table S2). Atomic coordinates and structure factors can be downloaded from the RCSB Protein Data Bank under accession codes 5N2B.

### 2.6. Computational Epitope Predictions

In order to identify possible antibody interaction sites on BPSL1626, we performed sequence- and structure-based analyses with seven different B and T cell epitope predictors. The following sequence-based predictors were employed: BCEPred [25], BepiPred (for our analyses, amino acids with a probability >50% were considered to be epitope residues [25], IEDB MHC-II binding predictor [26], and ANTIGENPro [27]. For predictions that are made with the T-cell IEDB MHC-II binding predictor, we selected the DRB1*09:01, DRB1*04:05, DRB1*15:02, and DRB1*03:01 human alleles, which are widespread in Thailand where melioidosis is endemic [17]. For structure-based predictions that are made with ElliPro [28], COBEPro [29], and REBELOT/BEPPE [30], we built gaps in the crystal structure of the dimer using the MODELLER framework [31] for both monomers. See Table S3 for more information on structure-based predictors.

### 2.7. Blood Sample Collection

Heparinized blood samples were collected from healthy donors at Khon Kaen University, Khon Kaen, Thailand with ethical approval by Khon Kaen University Ethics Committee for Human Research (Project no. HE470506). Seropositive and seronegative donors were defined on the basis of IHA titers of ≥1:40 and <1:40, respectively [32,33]. Recovery melioidosis patients were defined as individuals who had recovered from previously diagnosed melioidosis, as determined by isolation of *B. pseudomallei* from blood or tissues and who had completed antibiotic treatment.

### 2.8. Detection of IFN-γ Production in Whole Blood Culture

Heparinized whole blood from healthy donors (number of lymphocytes and monocytes were adjusted to 1.8 × 10^5^) were cultured in either: medium alone, 3 µg/mL phytohemagglutinin (PHA), paraformaldehyde (PFA)-fixed *B. pseudomallei* at 5.4 × 10^6^ CFU, or 10 µg/mL BPSL1626 protein for 48 h. IFN-γ levels in the culture supernatant were quantified using the human IFN-γ ELISA kit (BD Biosciences, US), following the manufacturer’s instructions.

### 2.9. Measurement of Human Plasma IgG Antibody Levels

The procedure for measuring plasma human IgG antibody levels was a modified version of a previously reported method [30,34]. Briefly, 96-well polystyrene plates (Nunc, Roskilde, Denmark) were coated overnight at 4 °C with carbonate coating buffer pH 9.6 (uncoated control), 10^7^ colony-forming units (CFUs) PFA-fixed *B. pseudomallei* or 10 µg/mL BPSL1626. Pre-coated plates were washed three times with washing buffer (phosphate buffer saline pH 7.4 (PBS) with 0.1% (*v*/*v*) Tween 20), and then blocked with 10% (*v*/*v*) fetal bovine serum (FBS) in PBS at room temperature for 2 h. Meanwhile, heparinized plasma samples were diluted at 1:300 with assay diluent (10% (*v*/*v*) FBS in PBS with 0.05% (*v*/*v*) Tween 20) before addition to the blocked ELISA plate and incubated at room temperature for 2 h. After five washes with washing buffer, the detection antibody mixture (1:10,000 biotinylated goat anti-human IgG, 1:5000 HRP conjugated streptavidin diluted in assay diluent) was added and incubated at room temperature for 1 h. Plates were washed seven times with washing buffer, prior to the addition of tetramethylbenzidine (TMB) substrate (BD Biosciences, Franklin Lakes, NJ, USA), incubating at room temperature for 10 min, before stopping the reaction with 2 N sulfuric acid. Absorbance readings were measured at a wavelength of 450/570 nm by ELISA. The results are reported as the Absorbance Index calculated by (O.D._test_ − O.D._uncoated_)/O.D. _uncoated_.

### 2.10. Statistical Analyses

Statistical analyses were carried out using Graphpad Prism version 6 (Graphpad, La Jolla, CA, USA). Statistical differences were determined using one-way ANOVA with Dunn’s multiple comparisons test for non-normal distribution data, or Tukey’s multiple comparisons test for normal distribution data. Data correlation was analyzed using the Pearson correlation.

### 2.11. Protein Microarray Assays

To test the immunoreactivity of BPSL1626, glass protein microarray slides were coated with copoly(DMA-NAS-MAPS) and treated, as previously reported [35]. BPSL1626 was spotted at the concentration of 1 mg/mL in PBS buffer. Microarrays were prepared using a non-contact sciFLEXARRAYER S3 (Scienion Co., Berlin, Germany) spotter. Printed slides were placed in a humid chamber and incubated overnight at room temperature. Microarrays were then blocked with 2% (*w*/*v*) BSA in PBS for 1 h, washed with water, and dried under a nitrogen stream.

Serum samples were diluted 1:100 in LowCross-Buffer^®^ (Candor, Wangen, Germany) and 40 μL were incubated for 60 min. Negative controls included blank arrays incubated only with the secondary antibody and with incubation buffer. The microarray slide was then rinsed three times with washing buffer (50 mM Tris-HCl pH 9, 0.25 M NaCl, 0.05% (*v*/*v*) Tween 20) and PBS and incubated with 40 μL of 1 μg/mL Cy-3 labeled goat anti-hIgG for another 60 min, followed by the same washing steps, as described above. Fluorescence was detected by a ProScanArray scanner (PerkinElmer, Boston, MA, USA) using 70% photomultiplier (PMT) gain and laser power. Fluorescence intensities were analyzed using the QuantArray software from PerkinElmer, corrected for spot-specific background. Values for replicate spots were averaged.

To assess seroreactivity against *B. cepacia* and *B. pseudomallei* infections, microarray tests were carried out on serum samples from CF patients harboring *B. cepacia complex* infections (N = 16) versus healthy serum samples (N = 10) (See details in [36]), and on melioidosis patients (N = 10) versus melioidosis recovery individuals (N =10) versus seronegative controls (N = 10), as judged by indirect hemagglutinin assay (IHA) antibody titers (Khon Kaen University and Srinakarin Hospital, Thailand). Student tests over the groups of samples were performed while using Prism 6 from GraphPad.

### 2.12. Rabbit Immunizations and Generation of Polyclonal Abs against BPSL1626

Polyclonal antibodies against BPSL1626 were produced by PRIMM Srl. (Naples, Italy), using standard company protocols. Briefly, two rabbits were immunized with four boosts at 0, 21, 28, and 35 days, before sacrifice at 39 days. Antibody titers were measured using ELISA, measuring the OD_490nm_ (corresponding to the signal produced by HRP-conjugated secondary anti-rabbit IgG) at various serum dilutions.

## 3. Results

### 3.1. In Silico Identification of BSL1626 as a Potential Antigen

Antigen candidates were selected via bioinformatics analysis of available genome sequences. First, proteins that are present in *B. pseudomallei* but not in the avirulent *B. thailandensis*, predicted to be non-cytoplasmic were identified and to be able to host the highest number of HLA epitopes. This generated a list of 104 candidates (Table S1). Prediction of HLA class I and class II epitopes indicated that almost all of the selected candidates contain fragments that are predicted to bind to HLA molecules with IC50 values lower than 50 nM.

Sequence conservation was assessed for all candidate proteins based on the initial set of eight *B. pseudomallei* proteomes. Remarkably, only four (BPSS2213, BPSS1498, BPSL1626, and BPSS1267) of the 104 proteins were fully conserved in the strains analyzed. The sequence variability of these four proteins with 349 *B. pseudomallei* strains is indicated in Figure S1. We selected BPSL1626 for further studies since it is annotated as a putative fimbrial protein with predicted extracellular localization, thus making it an attractive vaccine candidate. BPSL1626 sequence conservation was verified using 347 *B. pseudomallei* genomes. A multiple sequence alignment of the 347 orthologs revealed only two variable amino acids within the 176 positions of the alignment. Interestingly, the *Burkholderia mallei* ortholog (38% sequence identity) has also been identified as a candidate antigen in an independent reverse vaccinology study [37]. An additional search for orthologs, in 31 species of the *Burkholderia* genus, identified putative BPSL1626 orthologs in only two additional species: *B. oklahomensis* and *B. ubonensis*. Nevertheless, a further search for remote homologs found hits in all but four of the 31 species. Some of these could be also orthologs of BPSL1626.

### 3.2. 3D Structure Analyses of BPSL1626

**Figure 1:**
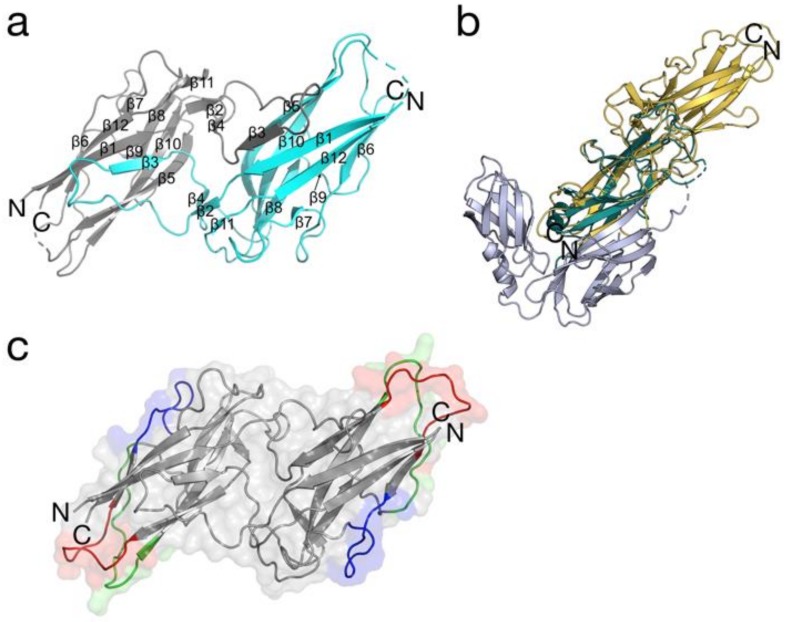
The crystal structure of BPSL1626. (**a**) Cartoon representation of the secondary structure organization chains A (grey) and B (turquoise) that comprise the BPSL1626 dimer. β-strands are numbered and the C- and N-termini of each chain are indicated. Gaps in the structure are indicated by dashed lines. (**b**) Structural superimposition of the BPSL1626 (gold ribbons) chain A with chain D of N-terminally truncated FimA (green ribbons) from *E. coli* strain K12 (PDB entry 4DWH). Two FimC chains are also shown (grey ribbons). (**c**) Location of computationally-predicted epitopes on the crystallographic BPSL1626 dimer. The three predicted epitope are highlighted in: red (epitope 81-LKNCGASTSGAT-92), blue (epitope 96-MGTTDSANPAA-106), and green (132-GSSSKAYTIAEGDNT-146) on each monomer. Surfaces are shown for the dimer, with appropriate shading for the epitope regions. (**a**) was generated using Pymol version 1.8. (**b**,**c**) were generated using Pymol 2.0.

Analysis of the crystal structure of BPSL1626 showed that two protein chains (A and B) are present in the asymmetric unit, forming dimers with symmetrically-related chains A and B, respectively (Figure 1a). Electron density is well-defined for residues 27 to 175 (chain A) and 28 to 175 (chain B). Overall density is better defined for chain A than chain B, and the latter contained two gaps (residues 84–90 and 142–143) correlating to flexible regions in comparison with one gap (residues 85–89) observed for the former.

Each BPSL1626 monomer comprises 13 β-strands, as calculated by PDBsum [38]. As previously reported, BPSL1626 is found on chromosome 1, in one of 13 gene clusters being predicted to be involved in the synthesis of type I fimbriae [39]. In agreement with other type I fimbrial proteins, BPSL1626 is organized as a dimer, with dimerization occurring via donor strand complementation, whereby the incomplete immunoglobulin-like fold of one chain is completed by the insertion of an N-terminal extension, contributing one β-strand (β3), from the adjacent chain (Figure 1a).

A Profunc (https://www.ebi.ac.uk/thornton-srv/databases/profunc/) structural homology search reveals that the BPSL1626 monomer shares the highest structural identity with the N-terminally truncated type 1 fimbrial protein A (FimA) from *E. coli* strain K12 (Chain D-PDB entry 4DWH) *rmsd* 1.65 Å (32.1% sequence identity; 104 aligned residues), solved in complex with the fimbrial chaperone (FimC) [40,41]. However, the dimer arrangement of BPSL1626 differs significantly to that of the FimA-FimC heterodimer (Figure 1b).

In contrast to previous reports that state that all of the pilus subunits contain an invariant disulfide bond that is introduced by the periplasmic disulfide oxidoreductase DsbA, BPSL1626 contains two highly conserved cysteines (C44 and C88), however they are located in distinct regions of the protein, and would not permit disulfide bond formation.

### 3.3. In Silico Epitope Predictions

Using the full-length sequence, BPSL1626 was evaluated as immunogenic by ANTIGENPro predictor (http://scratch.proteomics.ics.uci.edu/explanation.html), with a probability of 89% [27]. To identify precisely which kind of immune response that BPSL1626 can elicit, we performed distinct analyses with a series of in silico predictors (see Methods section). When considering the consensus between B-cell epitope predictions and the Matrix of Local Coupling Energies approach based on the three-dimensional (3D) structure (http://bioinf.uab.es/BEPPE/) [42], we identified a probable linear epitope 81-LKNCGASTSGAT-92 (red zone in Figure 1c). Furthermore, two other portions of the sequence showed a good consensus in sequence-based predictions, 96-MGTTDSANPAA-106 (blue zone in Figure 1c) and 132-GSSSKAYTIAEGDNT-146 (green zone in Figure 1c). See Supplementary Materials (Figure S2 and Table S3) for more details on the cut-off parameters and epitope predictors that were used. Notably, all three epitope candidates are located on the surface of the protein (Figure 1c), which is a necessary requirement for molecular recognition. Furthermore, two of those predicted interacting zones (epitopes 81–92 and 132–146) comprise unstructured loop regions of the crystal structure. This corroborates our hypothesis regarding the immunogenicity of the identified zones; these regions are not involved in the stabilization of the global structure, and thus have a likely role in inter-protein interactions.

### 3.4. Rabbit Immunizations with Recombinant BPSL1626

Antibody stimulation in rabbits was assessed by immunizing rabbits with recombinant BPSL1626, as described in the Methods. Immunization induced a strong immune response, as confirmed by the production of high antibody titres, confirmed via ELISA, in comparison with preimmune sera controls; at a serum dilution of 1/72,900, IgG antibody levels were >2500 fold higher than the preimmune sera controls (data not shown).

### 3.5. BPSL1626 Induces IFN-γ Production and Is Recognized by Human IgG Antibodies from Healthy Blood Samples Taken from Endemic Areas

**Figure 2:**
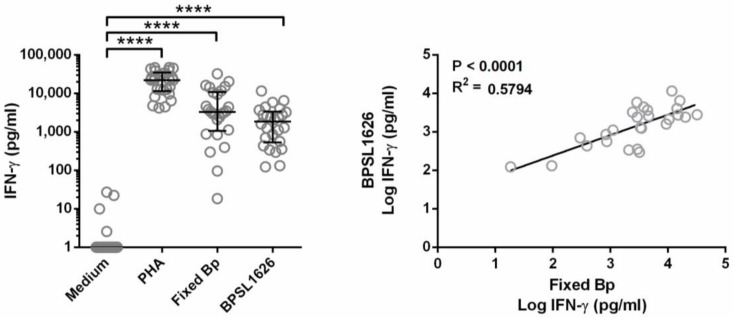
BPSL1626 induces IFN-γ production from healthy whole blood and correlates to the response from PFA-fixed *B. pseudomallei*. The left panel shows the IFN-γ released from whole blood from healthy donors, following incubation with medium alone, 3 μg/mL PHA, 5.4 × 10^6^ colony-forming units (CFU) paraformaldehyde (PFA)-fixed Bp, or 10 μg/mL BPSL1626 for 48 h, as described in the Methods section. IFN-γ levels in supernatants were quantified by ELISA; results are shown as individual scatter dot plot line at median with interquartile range. Statistical differences were determined using one-way ANOVA with Dunn’s multiple comparisons test or the Pearson correlation; **** *p* < 0.0001. The right panel shows the linear correlation between the Log IFN-γ production induced by BPSL1626 in comparison with PFA-fixed Bp, analyzed by the Pearson correlation test.

In order to confirm predicted BPSL1626 as an actual antigen, the ability of BPSL1626 to induce an innate immune response was ascertained by measuring its capacity to induce the production of IFN-γ, a key cytokine released by T-cells, Natural Killer cells and macrophages, and a known mediator of protective immunity against *B. pseudomallei* [43]. BPSL1626 was used to stimulate whole blood from seropositive healthy donors that were living in *B. pseudomallei* endemic areas. PHA, which is a polyclonal mitogen, was used as a positive control for IFN-γ activation, whereas PFA-fixed *B. pseudomallei* was used to represent the recall IFN-γ response upon stimulation with whole *B. pseudomallei*. Results show that BPSL1626 induces a significantly high level of IFN-γ production from whole blood cultures when compared to medium controls (*p* < 0.0001) (Figure 2).

Furthermore, data indicate that IFN-γ production induced by BPSL1626 and PFA-fixed *B. pseudomallei* stimulation are significantly correlated (*p* < 0.0001, R^2^ = 0.5794) (Figure 2). Such data imply that the BPSL1626 stimulation is linked to a recall immune response in healthy individuals living in endemic areas with continuous exposure to *B. pseudomallei*; this response corresponds to the level of response induced by the whole bacterium.

### 3.6. Probing the Human Antibody Response to BPSL1626 in Burkholderia Affected Individuals

**Figure 3:**
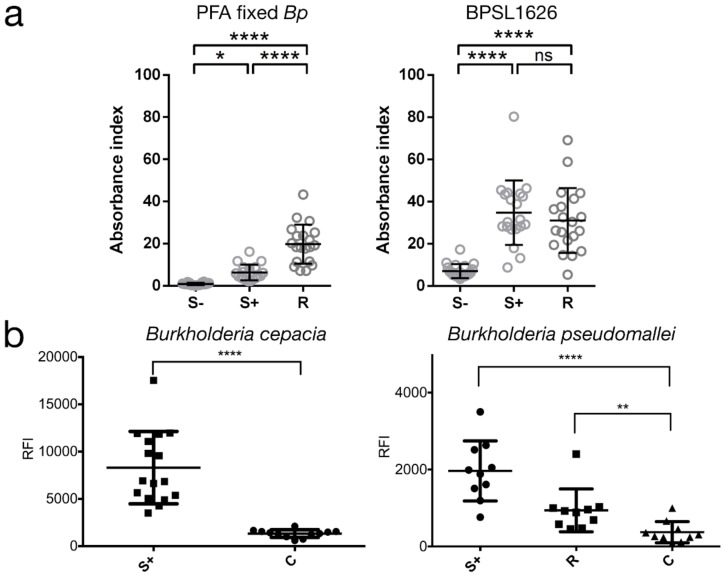
BPSL1626 is recognized by plasma IgG antibodies from seronegative, seropositive and melioidosis recovered individuals. (**a**) Plasma samples from seronegative (S−, N = 20), seropositive (S+, N = 20), or melioidosis recovered individuals (R, N = 20) were probed on uncoated, 10^7^ PFA fixed Bp, or BPLS1626 coated ELISA plates. Human IgGs bound to the ELISA plates were detected. The absorbance index was calculated by (O.D._test_ − O.D._uncoated_)/O.D._uncoated_. Results are shown as an individual dot plot line at mean with S.D. Statistical difference was determined using one-way ANOVA with Tukey’s multiple comparisons test; ns, non-significant, * *p* < 0.05, **** *p* < 0.0001; (**b**) Scatter plots reporting individual and mean immunoreactivity with S.D. of seropositive (S+) and control individuals. Results of the unpaired *t* Test for *B. cepacia complex* infection (left panel) and the unpaired *t* Test for *B. pseudomallei* infection (right panel). Seropositive patient groups are labeled as S+; recovery patients are labeled as R; seronegative control group is labeled as C. ns = not significant. Significant: *p* < 0.05; * = *p* < 0.05; ** = *p* < 0.01; *** = *p* < 0.001; **** = *p* < 0.0001.

To assess the ability of BPSL1626 to induce an antibody-mediated response, the levels of human IgG antibodies elicited in response to BPSL1626 were measured by both ELISA assay and protein microarrays using immune sera from seronegative (S−), seropositive (S+), or recovered melioidosis individuals (R). In ELISA assays, BPSL1626 was confirmed to induce an immune response, being strongly recognized by IgG antibodies from both seropositive and recovered melioidosis individuals, with reactivity being significantly higher than the seronegative controls (*p* < 0.0001) (Figure 3a). Immune sera recognition of BPSL1626 is comparable between seropositive and recovered melioidosis individuals (Figure 3a), suggesting that the antibody levels against BPSL1626 are not enhanced after recovery from infection. These findings were confirmed in protein microarray experiments using a different set of immune sera samples, as described in the Methods section (Figure 3b, right panel).

Furthermore, based on our previous findings that demonstrated that a panel of *B. pseudomallei* peptides, which were identified using a Structural Vaccinology (SV) approach, could also diagnose *B. cepacia complex* (*Bc*) infections in Cystic Fibrosis (CF) patients [36], recognition of BPSL1626 by serum IgGs from CF patients with *Bc* infections was investigated. A protein microarray displaying BPSL1626 was probed with 16 serum samples from CF patients presenting *Bc* infections, diagnosed by microbiological culture and MALDI-TOF spectrometry [36]. As a control group, seronegative serum samples from healthy donors were used (12 samples for the *B. cepacia* CF patient set, and 10 samples for melioidosis patients). The antigen-specific IgG content in each serum was evaluated by fluorescence detection using an anti-human IgG labeled with the Cy3 dye. The ability of BPSL1626 to distinguish between control and patient groups was evaluated by performing the unpaired *t* Test (significant if *p* values < 0.05) on the measured protein-specific fluorescence signals (see Figure 3b, left panel). BPSL1626 resulted to be effective in capturing patient antibodies that were elicited in response to both *B. cepacia* and *B. pseudomallei* infections. As previously mentioned, there is extremely low sequence conservation of the BPSL1626 gene among non-*pseudomallei Burkholderia* species, suggesting that the 3D structure impacts heavily on epitope-antibody recognition.

## 4. Discussion

Since the first application of reverse vaccinology methods for the development of the Men B vaccine, in silico antigen identification has been carried out on different pathogens, including *Mycobacterium tuberculosis*, *Chlamydia trachomatis* and *Chlamydia pneumoniae*, and uropathogenic *E. coli* [44,45,46,47].

Using a RV approach, we predicted that 104 potential *B. pseudomallei* antigens to be transferred to further experimental validation. To this aim, we analyzed BPSL1626, a RV-predicted antigen selected in the context of a medium-throughput reverse and structural vaccinology study, based on its predicted extracellular location and its prediction as a correctly-folded and soluble protein, following a *B. pseudomallei* genomic ORF-filtering library study [48]. To date, other than genomic analyses that imply its involvement in synthesis of Type I fimbria [39], the functional role of BPSL1626 remains to be characterized.

BPSL1626 was heterologously expressed in *E. coli* and was found to be recognized by serum IgGs from seropositive and convalescent melioidosis patients, in comparison with healthy controls. Such findings indicate that a previous immune response to this antigen has been triggered, although there is no enhanced immune response following recovery from melioidosis. Previous studies have underlined the presence of conserved epitopes between *B. pseudomallei* and *B. cepacia* antigens [49], and a peptide microarray presenting *B. pseudomallei* epitope peptides was shown to be able to diagnose *B. cepacia* infections in Cystic Fibrosis (CF) patients [36]. Indeed, despite the low sequence identity between BPSL1626 and its *B. cepacia* ortholog, BPSL1626 was recognized by serum antibodies from CF patients, underlining the importance of epitope 3D conformation for antibody binding and cross-recognition.

The capacity to stimulate innate and adaptive immune responses is crucial for a potential melioidosis vaccine; therefore, we subsequently probed the ability of BPSL1626 to stimulate T-cells [50]. BPSL1626 was found to trigger the release of IFNγ from whole blood from seropositive healthy donors living in *B. pseudomallei* endemic areas, i.e., in continuous environmental contact with the pathogen. Such activation was found to be correlated to a recall immune response, corresponding to the level of recall response induced by the whole bacterium.

Previous SV studies, which were carried out on other *B. pseudomallei* antigens, have demonstrated that antigenic epitopes can be accurately predicted using sequence- and structure-based computational epitope prediction methods [51]. By ‘removing’ epitopes from their recombinant antigen and synthesizing them as isolated peptides or peptidomimetics, such epitopes may acquire improved immunological properties, as observed for a neutralizing epitope from the BPSL2765 antigen [34].

Type I fimbria are long filamentous structures, comprising thousands of copies of the fimbrial subunit A (FimA) that assemble to form the pilus rod, enabling pathogenic bacteria to adhere to host cells. FimD anchors the pilus to the outer membrane and a flexible tip fibrillum is located at the end of the pilus rod, comprising single or multiple copies of the minor subunits FimG and two FimF subunits, and a single copy of FimH. FimF is bound to the FimC chaperone, which binds to unfolded fimbrial components in the periplasm, and mediates their correct folding and ushers them to outer membrane protein [52].

The 3D structures of the diverse fimbrial components that have been solved to date, present an incomplete immunoglobulin-like fold, whereby the missing β-strand is contributed by a polypeptide segment of a distinct fimbrial subunit, and may be donated by the FimC chaperone, or by an adjacent pilus subunit [52]. BPSL1626 is no exception to this rule and it presents the same conserved Type I fimbria fold, with two short β strands from one monomer donating a shorter-than-usual β-strand (β3) that completes the immunoglobulin-like domain of the opposing monomer.

In order to locate potential antigenic regions of the structure, we applied sequence- and structure-based epitope predictors to the BPSL1626 crystal structure, and located three key epitopes for further study as diagnostic and or therapeutic molecules. Predicted epitopes are currently being synthesized as isolated peptides and will be tested for their immunoreactivity and as components for vaccine formulations in future experiments.

In conclusion, when considering the promising immunoreactivity of the full length protein and the immunoreactivity demonstrated by peptides generated via a protocol similar to the one that we applied, we propose that the structural and functional characterization of BPSL1626 presented here may constitute a valid basis for the design of multicomponent vaccine candidates to be used against *B. pseudomallei* infections.

## 5. Conclusions

Starting from a list of 104 in silico predicted *B. pseudomallei* antigens, we confirm that candidate antigen BPSL1626 can induce antibody and T-cell responses in melioidosis patients. Furthermore, BPSL1626 proved serodiagnostic for both *B. pseudomallei* and *B. cepacia* infections in melioidosis and CF patients, respectively. Furthermore, we present the crystal structure of BPSL1626 that supports its predicted role in Type I fimbria formation. With the scope of future antigen redesign, we also predicted three antigenic regions while using both sequence- and structure-based epitope prediction methods. Our findings imply that BPSL1626 and its epitopes should be further studied as a potential protective *B. pseudomallei* components.

## Supplementary Materials

The following are available online at http://www.mdpi.com/2073-4468/7/3/26/s1, Table S1: List of 104 potential *B. pseudomallei* antigens, identified using a reverse vaccinology approach; Table S2: Data collection and refinement statistics for the crystal structure of BPSL1626; Table S3: Brief description of the structure-based epitope prediction methods adopted and applied to the dimer structure of BPSL1626; Figure S1: Variability profile of BPSS2213, BPSS1498, BPSS1267 and BPSL1626 generated from multiple protein sequence alignments using 349 *B. pseudomallei* strains; Figure S2: Raw epitope prediction data made with structure- and sequence-based predictors.
