# Supplementary material for: BPSL1626: Reverse and Structural Vaccinology Reveal a Novel Candidate for Vaccine Design against Burkholderia pseudomallei
Source: Antibodies (Basel). 2018 Jul 19;7(3):26. doi: 10.3390/antib7030026 (PMC6640674; doi:10.3390/antib7030026)
Supplement: Supplementary file 1 [file antibodies-07-00026-s001.zip › SI_corr/SI_BPSL1626_corr.pdf]

Supplemental Materials:

Table S1: List of 104 potential *B. pseudomallei* antigens, identified using a reverse vaccinology approach (See Table attached as separate excel file).

Table S2. Data collection and refinement statistics for the crystal structure of BPSL1626.

Table S3. Brief description of the structure-based epitope prediction methods adopted and applied to the dimer structure of BPSL1626.

Figure S1. Variability profile generated from multiple protein sequence alignments using 349 *B. pseudomallei* strains.

Figure S2. Raw epitope prediction data made with structure- and sequence-based predictors.

Supplementary references

Table S2. Data collection parameters and refinement statistics for the crystal structure of BPSL1626. Data were collected on a single crystal. $R_{merge} = \sum|I-(I)/\sum I \times 100$, where I is the intensity of a reflection and *(I)* is the average intensity; Values in parentheses represent data belonging to highest resolution shells (2.0-1.9Å).

	**BPSL1626**
**Data collection**	
Space group	$C222_1$
Cell dimensions	
a, *b*, *c* (Å)	91.9 102.2 72.4
α, β, γ (°)	90 90 90
Resolution (Å)	40-1.9 (2.0-1.9)
R_{merge}	0.041 (0.479)
$I / \sigma I$	31.6 (4.4)
Completeness (%)	99.9 (100)
Redundancy	7.4 (7.5)
**Refinement**	
Resolution (Å)	34-1.9
No. unique reflections	27236 (3930)
R_{gen} / R_{free}	20.9/23.7
No. atoms	
Protein	1960
Ethylene glycol	110
Water	98
B-factors (Å^2)	
Protein	42.6
Ethylene glycol	69.4

Water	40.3
R.m.s. deviations	
Bond lengths (Å)	0.002
Bond angles (°)	0.493
Ramachandran Plot (%)	
Favored Regions	96.7
Allowed Regions	100

Table S3. Brief description of the structure-based epitope prediction methods adopted and applied to the dimer structure of BPSL1626.

**Predictor**	**Description**
ElliPro	Structure-based B-cell epitope predictor that assigns a protrusion index (*i.e.,* the percentage of every residue that are inside an ellipsoid built around the protein) to each residues. Amino acids that have a protrusion index greater that the threshold are identified [1].
COBEPro	Structure-based continuous B-cell epitope predictor that implements a Support Vector Machine (SVM) to identify possible epitope fragments. It assigns a probability to epitope fragments using the same scores obtained from the machine learning identification procedure. An epitope residue has a probability >75%.
REBELOT/BEPPE	Structure based B and T cell epitope prediction via energy decomposition analysis [2]. BEPPE exploits the Matrix of Local Coupling (MLCE) method to identify antigenic regions [3].

Figure S1. The variability of BPSS2213, BPSS1498, BPSS1267 and BPSL1626 in 349 *B. pseudomallei* strains was analyzed by calculating the Shannon Entropy for each residue at a certain position using the Protein Variability Server (PVS) [4-5]. Higher Shannon Entropy values indicate higher variability in that particular position in the alignment. Amino acid positions are represented according to the K96243 sequence numbering.

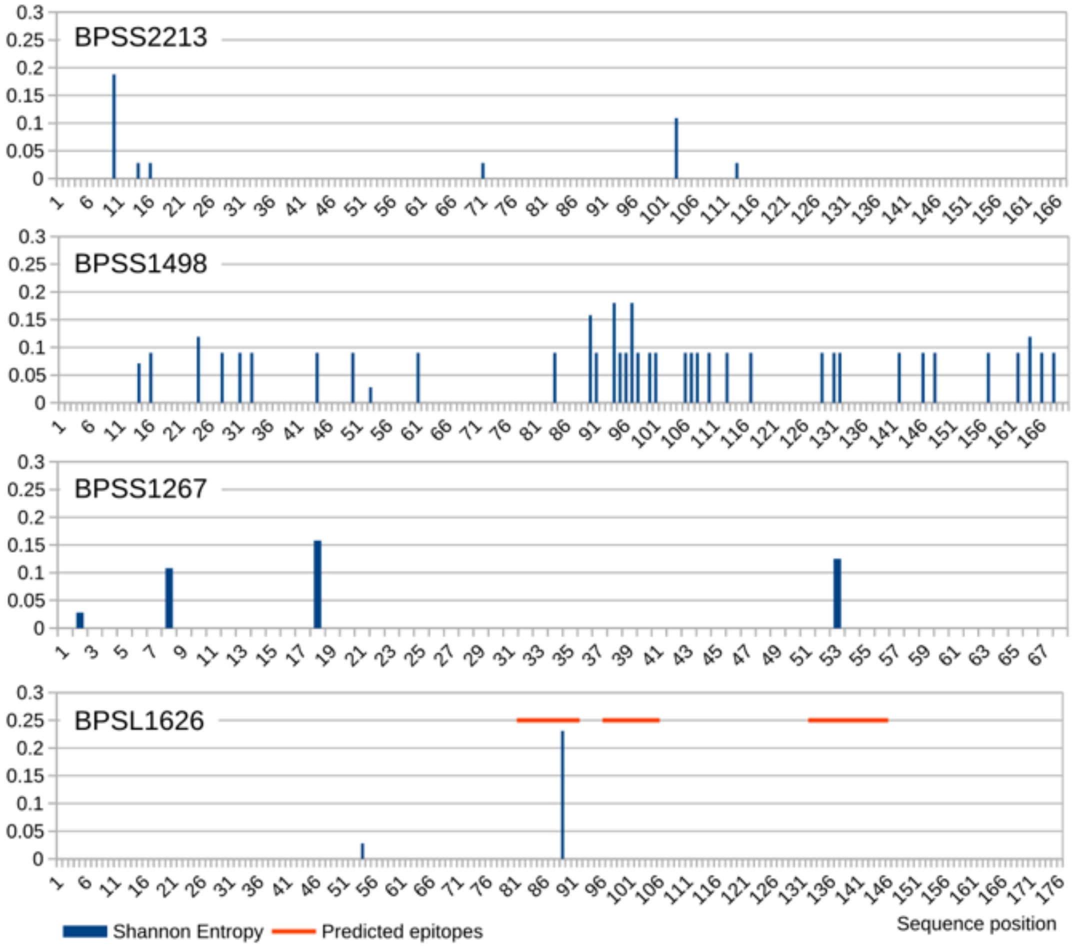

Figure S2. Raw epitope prediction data made with structure- and sequence-based predictors. Predicted epitope residues, found by all predictors used are marked with an "E" in the sequence. Residues marked in red refer to flexible areas in the crystal structure that were not built due to lack of electron density and thus were reconstructed using MODELLER. Programs renumber the PDB sequence, thus residue 1 = 27, 149 = 175 in the Uniprot sequence (Uniprot code Q63UH6).

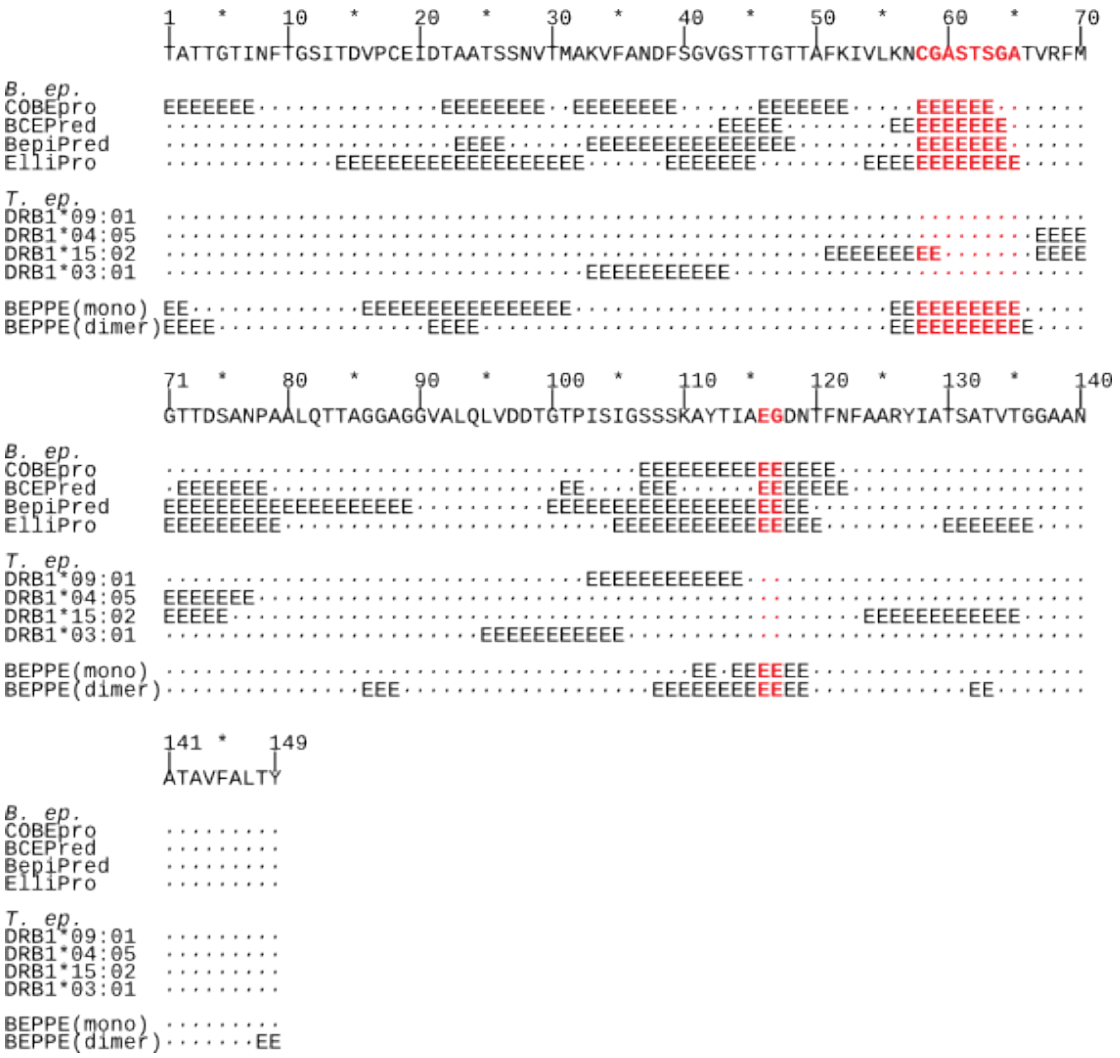

Supplementary references:

[1] Ponomarenko, J.; Bui, H.H.; Li, W.; Fusseder, N.; Bourne, P.E.; Sette, A.; Peters, B. Ellipro: A New Structure-Based Tool for the Prediction of Antibody Epitopes. *BMC Bioinform.* **2008**, *9*, 514

[2] Peri C.; Solé O.C.; Corrada D.; Gori A.; Daura X.; Colombo G. Prediction of Antigenic B and T Cell Epitopes via Energy Decomposition Analysis: Description of the Web-Based Prediction Tool BEPPE. *Methods Mol Biol*. **2015**,*1348*, 13-22.

[3] Scarabelli, G.; Morra, G.; Colombo, G. Predicting Interaction Sites from the Energetics of Isolated Proteins: A New Approach to Epitope Mapping. *Biophys. J.* **2010**, *98*, 1966–1975.

[4] Shannon, C.E. *The mathematical theory of communication*. *The Bell system Technical Journal* **1948**, *27*, 379-423 & 623-656.

[5] Garcia-Boronat M.; Diez-Rivero C.M.; Reinherz E.L; Reche P.A. PVS: a web server for protein sequence variability analysis tuned to facilitate conserved epitope discovery. *Nucleic Acids Res* **2008,** *36*, W35-41.
